# Evaluation of the effects of medium‐term (57‐day) omeprazole administration and of omeprazole discontinuation on serum gastrin and serum chromogranin A concentrations in the horse

**DOI:** 10.1111/jvim.16795

**Published:** 2023-06-30

**Authors:** Bethanie Clark, Catherine Steel, Jessica Vokes, Jack R. Shan, Kristene Gedye, Amy Lovett, B. W. Sykes

**Affiliations:** ^1^ School of Agriculture and Food Sciences University of Queensland Gatton Queensland Australia; ^2^ Department of Veterinary Clinical Service The Hong Kong Jockey Club Hong Kong Hong Kong; ^3^ School of Veterinary Science Massey University Palmerston North New Zealand

**Keywords:** equine, ESGD, hypergastrinemia, rebound gastric hyperacidity, stomach

## Abstract

**Background:**

Rebound gastric hyperacidity (RGH) secondary to hypergastrinemia has been suggested to contribute to the rapid recurrence of equine squamous gastric disease (ESGD) in horses after discontinuation of omeprazole.

**Hypothesis/Objectives:**

To evaluate changes in serum gastrin and chromogranin A (CgA) concentrations in response to medium‐term (57‐day) omeprazole treatment and after omeprazole discontinuation.

**Animals:**

Fourteen mature Thoroughbred racehorses in simulated race training.

**Methods:**

Horses received 2.28 g of oral omeprazole PO q24h for 57 days within a 61‐day period, excluding a withholding period applied mid‐protocol during which treatment was stopped as part of a concurrent study. Serum samples were collected on day 0 before omeprazole treatment, on day 1 of each week of the treatment period, and for an additional 5 weeks after discontinuation of treatment. Serum gastrin and CgA concentrations were analyzed using radioimmunoassay (RIA) and ELISA, respectively.

**Results:**

Median serum gastrin concentrations increased 2.5‐fold from baseline to day 7 (*P* < .001) but did not increase further during the omeprazole treatment period. Median serum gastrin concentrations returned to baseline within 2 to 4 days after administration of the last dose of omeprazole. No effect of treatment or discontinuation was seen in serum CgA concentrations.

**Conclusions and Clinical Importance:**

Serum gastrin concentrations increased in response to omeprazole treatment but returned to baseline within 2 to 4 days after the last dose of omeprazole. No effect of treatment or discontinuation was seen in serum CgA concentrations. Our results do not support the use of tapering protocols in horses.

**Abbreviations**
CgA: chromogranin A
ECL: enterochromaffin‐like
ESGD: equine squamous gastric disease
PPI: proton pump inhibitor
RGH: rebound gastric hyperacidity
RIA: radioimmunoassay
RWP: recommended withholding period

## 1 INTRODUCTION

Concern has grown about the overuse and safety of proton pump inhibitor (PPI) treatment in both humans and horses.^1^, ^2^, ^3^ One concern is the reccurrence of disease after the discontinuation of treatment caused by rebound gastric hyperacidity (RGH).^1^, ^3^

Gastrin contributes to the regulation of gastric acid secretion by direct effects on parietal cells and by increasing enterochromaffin‐like (ECL) cells.^4^, ^5^ Gastrin production normally is regulated by gastric pH, with production inhibited by a decrease in gastric fluid pH and direct effect of somatostatin release from D‐cells.^6^ In humans, acid suppression by PPI administration results in a loss of negative feedback on gastrin, resulting in increased gastrin production and hypergastrinemia. Furthermore, gastrin has a positive trophic effect on ECL cell density.^5^, ^7^ Histamine and ECL cells play important roles in the stimulation of acid secretion by gastrin, and hypergastrinemia, coupled with increased ECL cell density, increases the acid secretory capacity of the stomach, leading to RGH after omeprazole discontinuation in humans.^1^ Increased ECL cell density is evaluated by serum chromogranin A (CgA).^8^, ^9^ Both increased serum gastrin and CgA concentrations have been observed in rats and humans during PPI treatment, indicating the presence of hypergastrinemia and ECL cell hyperplasia.^7^, ^9^, ^10^, ^11^, ^12^

Rebound gastric hyperacidity has been reported to result in the rapid reccurrence of acid‐related symptoms in 47% to 58% of human patients.^1^, ^10^, ^11^, ^13^ Serum gastrin concentrations have been reported to double within 14 days of omeprazole treatment in horses.^2^ The clinical relevance of this finding is unclear, but a recent study comparing withholding periods for omeprazole treatments in racing Thoroughbreds reported an equine squamous gastric disease (ESGD) prevalence of 83% in horses after a “2 clear days” recommended withholding period (RWP) for omeprazole, which was an increase from 25% of horses with ESGD before the RWP.^14^ These results suggest that RGH might contribute to the rapid reccurrence of disease.^13^, ^14^

Our aims were to evaluate the effects of medium‐term (57‐day) omeprazole administration and its abrupt discontinuation on serum gastrin and CgA concentrations in the horse. It was hypothesized that omeprazole treatment and abrupt discontinuation would result in the development of hypergastrinemia and increased ECL cell density.

## 2 MATERIALS AND METHODS

### 2.1 Animals

Fourteen retired Thoroughbred racehorses used for jockey training were sourced from the Hong Kong Jockey Club's Conghua jockey training school. All horses were geldings between 8 and 14 years of age. Horses were included if they were normal on clinical and lameness examination, and the trainer considered the horse to be suitable for a conditioning program over a 10‐week period. Horses were excluded if they had received omeprazole or another acid suppressive drug in the 2 months before enrollment in the study. Horses underwent gastroscopic examination on day 0, day 28 (pre‐withhold), day 31 (post‐withhold), day 59 (pre‐withhold), and day 62 (post‐withhold) as part of a simultaneous study.^14^ Gastroscopy results were not required for inclusion or exclusion into this study and the results are reported elsewhere.^14^

Throughout the study, animals remained in their usual stable environment and were fed their usual diet of timothy hay (7 kg each morning) and alfalfa hay (5 kg each afternoon), commercial complete feeds (1.8 kg Connolly's Red Mills 10% [Connolly's Red Mills, Goresbridge, Ireland] and 0.45 kg of 14% Connolly's Red Mills 14% [Connolly's Red Mills, Goresbridge, Ireland] twice daily), 0.5 kg fermented alfalfa (FiberProtect, FiberFresh, Reporoa, New Zealand) each morning, 1.3 kg ricebran (EquiJewel, Kentucky Equine Research, Versailles, Kentucky) twice daily, and 70 g corn oil twice daily. All horses underwent a standard 10‐week conditioning program for race preparation under the same trainer throughout the study period. The study was approved by the Ethics Committee of the Hong Kong Jockey Club (protocol code ERC/031/2021).

### 2.2 Drug administration

From day 1 to day 61 (except for RWPs), horses received 2.28 g (4.4‐5.3 mg/kg for approximately 450‐550 kg horses) of a commercially available, buffered paste^15^ omeprazole formulation PO q24h at 4 am. To maximize absorption and activation, the omeprazole was administered 30 minutes before morning feeding with 0.5 kg of fermented alfalfa (FiberProtect, FiberFresh, Reporoa, New Zealand).^16^ Feeding was performed pre‐exercise during both treatment and the RWPs.

During a concurrent study, horses were subject to 2 RWPs (“2‐clear‐days” and “not on race day”). Fourteen horses were allocated into 2 equal groups at enrollment. Group A (n = 7) were subjected to the “2 clear days” withhold at the end of week 4 of treatment (withheld for days 29‐31 inclusive) and the “not on race day” withhold at the end of week 8 of treatment (treatment ended on day 61). Group B horses (n = 7) were subjected to the “not on race day” withhold at the end of week 4 of treatment (withheld for day 31 only) and to the “2 clear days” withhold at the end of week 8 of treatment (treatment ended on day 59). Each horse was treated with omeprazole for 57 days throughout the 61‐day period.^14^

### 2.3 Serum gastrin and CgA sampling and analysis

Baseline blood samples were collected on day 0, before omeprazole treatment. After commencement of omeprazole treatment, blood samples were taken on days 7, 14, 21, 28, 35, 42, 49 and 56 of the treatment period. After discontinuation of omeprazole treatment, blood samples were taken for an additional 5 weeks, on days 63, 70, 77, 84 and 91. All blood samples were taken 5 minutes before the morning feeding; 10 mL of blood was collected by jugular venipuncture directly into a plain serum tube. The samples were separated and frozen within 4 hours and stored frozen at −20°C at the Hong Kong Jockey Club. The samples were transported frozen to Massey University (New Zealand) where they were stored at −20°C until analysis.

Serum gastrin concentrations were analyzed using gastrin radioimmunoassay (RIA) at an external commercial laboratory, Canterbury Health Laboratories (Canterbury, New Zealand), using a previously validated method of measuring gastrin in equine serum.^17^ The test was performed to commercial standards and internal calibration (data not available). Serum CgA concentrations were analyzed using commercially available ELISA kits (horse CgA [CHGA] ELISA Kit, MyBioSource Inc, San Diego, California [capture antibody: mouse, monoclonal; immunogen: recombinant full‐length horse CgA and detection antibody: rabbit, polyclonal; immunogen: recombinant fragment corresponding to aa 116‐362 of horse CgA]) according to the manufacturer's instructions at Massey University, New Zealand. Standard concentration gradients (500, 250, 125, 62.5, 31.25, 15.6 ng/mL) and blanks provided in the ELISA kits were utilized according to the manufacturer's instructions to generate a standard curve for each plate, from which CgA concentrations were calculated. The limit of detection for the CgA ELISA was 15.6 to 500 ng/mL, as determined and provided by the manufacturer. Although an ELISA to measure serum CgA in horses has been developed previously,^18^ CHGA has not yet been validated for use in horses.

### 2.4 Data analysis

For both serum gastrin and CgA concentrations, data were collated into a Microsoft Excel spreadsheet (Microsoft Excel, Microsoft Corporation, Redmond, Washington). The Shapiro‐Wilks test was used to test for normality. The data were not normally distributed and could not be normalized by transformation, hence a nonparametric test was selected. The Kruskal Wallis test for multiple comparisons was used to assess treatment effect over time, in comparison to baseline measurements. Data are presented as median and interquartile range [IQR] for each serum concentration. The post hoc Dunn's test using a Bonferroni corrected alpha was used to calculate a corrected *α* for multiple comparisons to determine significance between weekly median serum gastrin concentrations and median serum CgA concentrations. Statistical tests were performed using an online calculator (https://www.statskingdom.com, Statistics Kingdom, Melbourne, Australia). Significance was defined as *P* < .001, according to the Bonferroni corrected alpha for multiple comparisons. Variables with *P*‐values between <.001 and <.05 are highlighted in the results to allow comparison between pre‐ and post‐correction for multiple comparisons.

The magnitude of intra‐run variability for the CgA ELISA was determined by calculating the coefficient of variation of 9 sample replicates run within the same plate, using Microsoft Excel (Microsoft Excel, Microsoft Corporation, Redmond, Washington). Inter‐run variability for the CgA ELISA was determined by calculating the coefficient of variation of sample replicates across 3 different plates using Microsoft Excel (Microsoft Excel, Microsoft Corporation, Redmond, Washington).

## 3 RESULTS

### 3.1 Animals

All 14 horses completed the study and omeprazole was administered as planned each day, including a lack of administration on RWPs (days 29‐31 and day 61 [group A] or day 31 and days 59‐61 [group B]). Each horse received 57 daily doses of omeprazole, the final dose was given on day 58 (group B) or day 61 (group A). Gastroscopy results are presented elsewhere.^14^

### 3.2 Gastrin

A full data set was available for serum gastrin concentrations. The post hoc Dunn's test to correct for multiple comparisons using a Bonferroni corrected alpha calculated a corrected *α* of .0005 to determine significance among weekly median serum gastrin concentrations.

**FIGURE 1:**
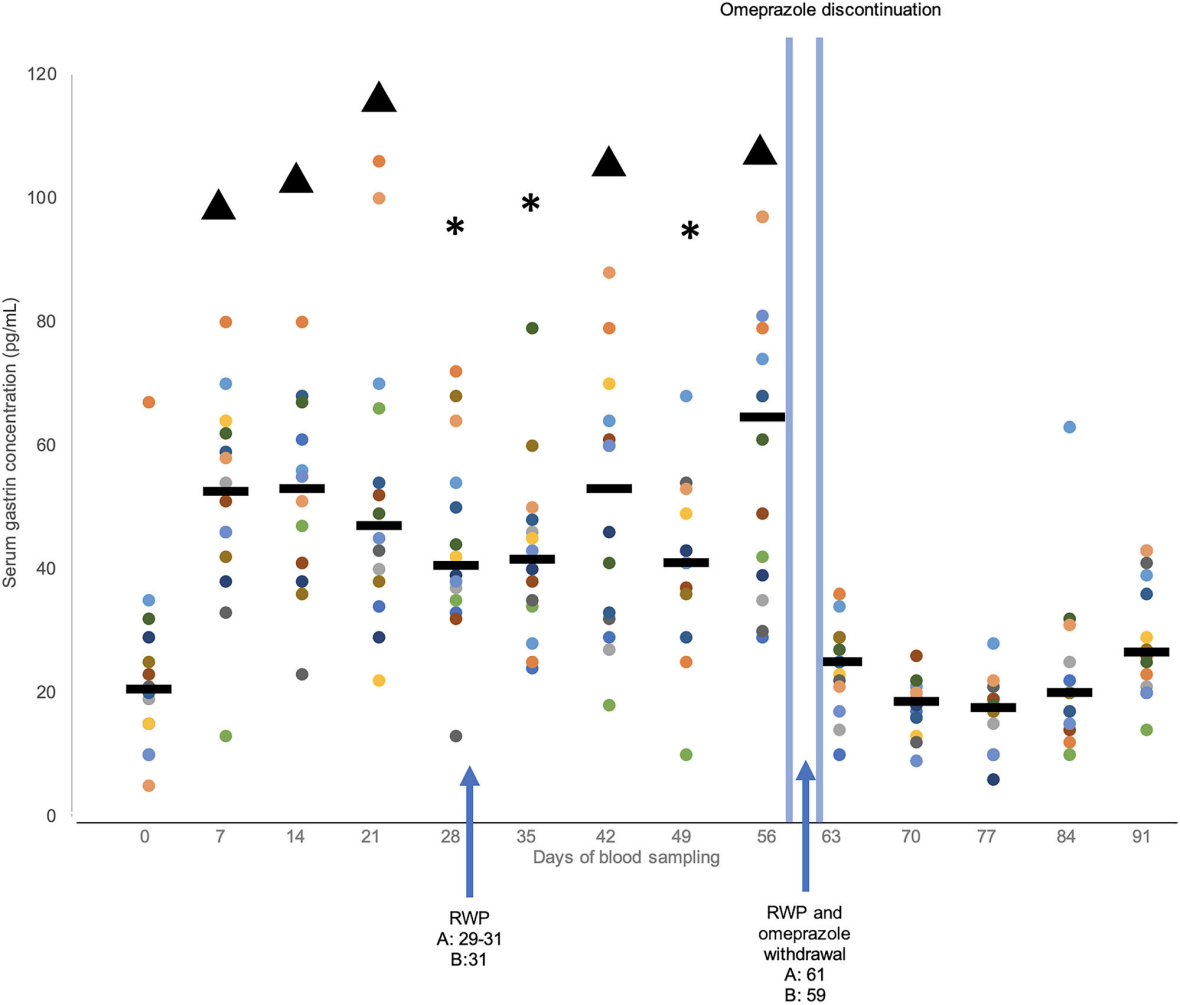
Radioimmunoassay analysis of fasted serum gastrin concentrations for 14 Thoroughbred racehorses and medians for each day. Horses were subject to omeprazole treatment from days 1 to 61, except for recommended with holding periods (RWP) at the end of week 4 and 8 indicated by the arrows. Day 0 are baseline measurements and days 63 to 91 are post omeprazole treatment. *P < .05 and ^*P* < .0001 to indicate differences from baseline (day 0) measurements before and after correction for multiple comparisons, respectively.

Median serum gastrin concentrations (pg/mL [IQR]) increased 2.5‐fold from baseline on day 0 (20.5 [15, 28]) to day 7 (52.5 [43, 61.25]) after the beginning of omeprazole treatment (*P* < .001). Serum gastrin concentration remained increased at each sample time (day 7 [52.5 (43, 61.25)]; day 14 [53 (41, 65.5)]; day 21 [47 (38.5, 63)]; day 28 [40.5 (35.5, 53)]; day 35 [41.5 (34.25, 47.5)]; day 42 [53 (32.25, 63.25)]; day 49 [41.0 (36.25, 47.5)]) until day 56 [64.5 (39.75, 77.75); *P* < .001]; Figure 1). After correction for multiple comparisons, days 7, 14, 21, 42 and 56 remained significantly higher than baseline serum gastrin concentrations (*P* < .001). Once increased, serum gastrin concentrations did not differ during omeprazole treatment between days 7 and 49 inclusive (*P* > .05). Serum gastrin concentration increased between days 49 and 56 (*P* = .02), but after correction for multiple comparisons, this difference was not significant (*P* > .001).

Median serum gastrin concentration did not differ significantly from baseline (20.5 [15, 28]) for all days after discontinuation of treatment on days 59 and 61 (day 63 [25.0 (21.25, 28.5); *P* = .74], day 70 [18.5 (16, 20.75); *P* = .37], day 77 [17.5 (11.25, 19); *P* = .26], day 84 [20.0 (15.5, 24.25); *P* = .87], and day 91 [26.5 (21.5, 38.25); *P* = .24]). Median serum gastrin concentrations decreased significantly when days 7 (52.5 [43, 61.25]) and 56 (64.5 [39.75, 77.75]), the first and last weeks of omeprazole treatment, were compared to day 63 (25.0 [21.25, 28.5]), day 70 (18.5 [16, 20.75]), day 77 (17.5 [11.25, 19]), day 84 (20.0 [15.5, 24.25]), and day 91 (26.5 [21.5, 38.25]; *P* < .001).

### 3.3 Chromogranin A

Serum CgA concentration results were below the limit of detection (<15.6 ng/mL) for 11/196 (5.6%) samples (horse 1—day 7; horse 13—days 0, 7, 14, 21, 28, 35, 42, 77, 84, and 91). These data points were excluded from analysis. The post hoc Dunn's test to correct for multiple comparisons using a Bonferroni corrected alpha calculated a corrected *α* of .0005 to determine significance between weekly median serum CgA concentrations. Standard curve correlations (*R* ^2^) all were >0.98. Intra‐run and inter‐run variability were 17.5% and 64.5%, respectively.

**FIGURE 2:**
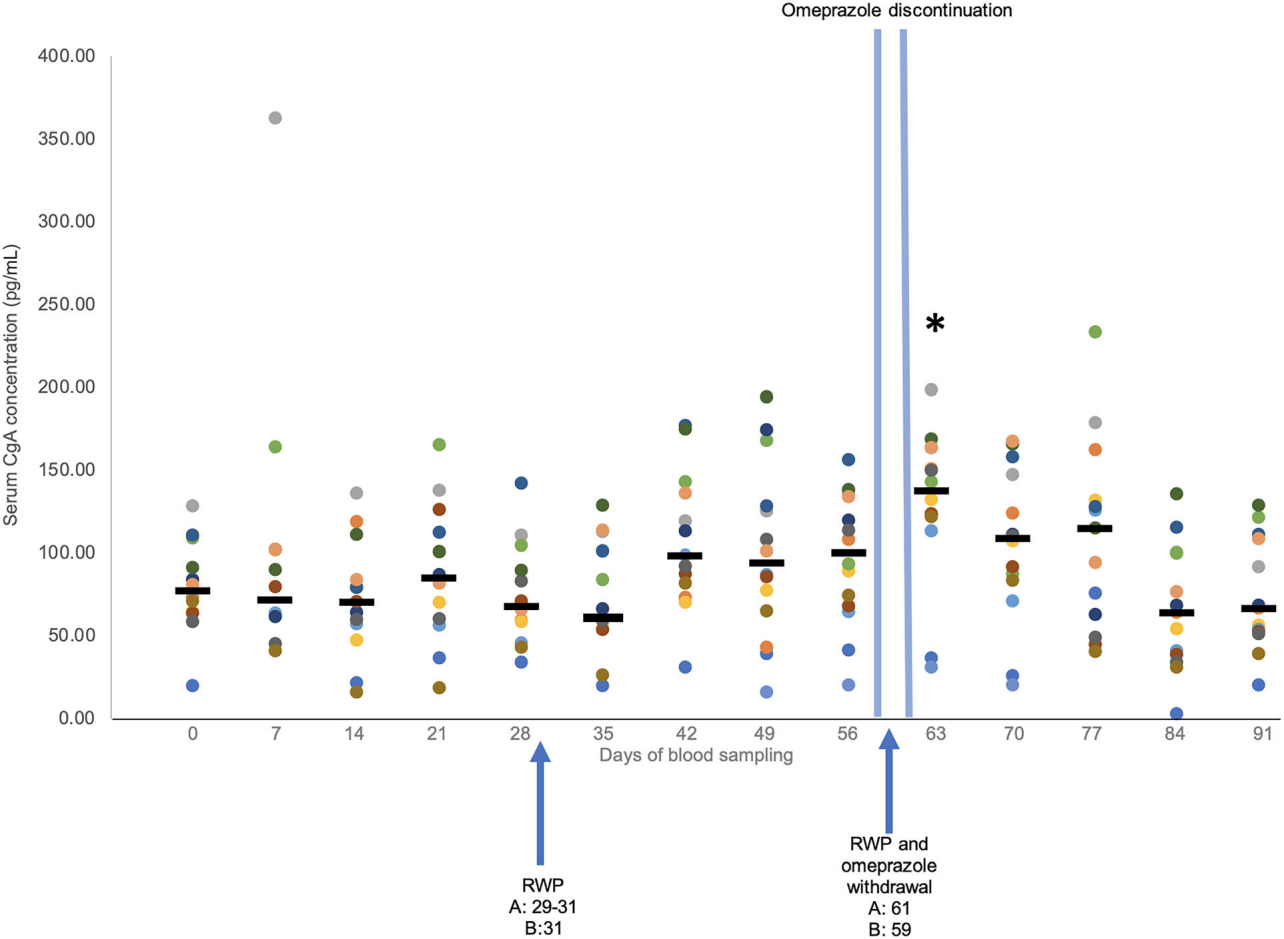
Enzyme‐linked immunosorbent assay analysis of fasted serum CgA concentrations for 14 Thoroughbred racehorses and medians for each day. Horses were subject to omeprazole treatment from days 1 to 61, except for recommended withholdingperiods (RWP) at the end of week 4 and 8 indicated by the arrows. Day 0 are baseline measurements and days 63 to 91 are post omeprazole treatment. *P < .05 to indicate differences from baseline (day 0) measurements.

After corrections for multiple comparisons, median serum CgA concentrations (ng/mL [IQR]) did not change significantly from baseline on day 0 (77.18 [64.83, 90.93]) to day 7 (71.75 [62, 101.73]; *P* = .84), day 14 (70.28 [56.78, 84.23]; *P* = .64), day 21 (84.93 [60.53, 112.38]; *P* = .7), day 28 (67.98 [58.53, 89.58]; *P* = .68), day 35 (60.93 [53.98, 101.5]; *P* = .57), day 42 (98.83 [81.83, 136.17]; *P* = .11), day 49 (94.08 [68.21, 127.83]; *P* = .21) and day 56 (100.5 [69.42, 130.29]; *P* = .27) during the omeprazole treatment period (Figure 2). After the last doses of omeprazole on days 59 or 61, median serum CgA concentration on day 63 (137.58 [122.42, 160.21]) was 1.8‐fold higher than on day 0 (77.18 [64.83, 90.93]; *P* = .001). After correction for multiple comparisons, day 63 was not significantly different from baseline nor were day 70 (108.67 [84.29, 141.46]; *P* = .14), day 77 (115 [63.05, 131.5]; *P* = .17), day 84 (64.14 [38.86, 99.86]; *P* = .38) and day 91 (66.95 [52.62, 108.48]; *P* = .66).

## 4 DISCUSSION

Our aims were to evaluate the effects of medium‐term (57‐day) omeprazole administration and the discontinuation of omeprazole treatment on serum gastrin and CgA concentrations in the horse. The hypothesis that omeprazole treatment and abrupt discontinuation would influence serum gastrin concentrations was proven, whereas the hypothesized change in serum CgA concentration was not clearly identified. We identified an increase in serum gastrin concentration within 7 days of initiation of omeprazole treatment and the return to baseline within 2 to 4 days of administration of the last dose of omeprazole. We did not identify a clear change in serum CgA concentrations in response to omeprazole treatment.

Our study consisted of each horse receiving 2.28 g omeprazole PO for 57 days, double the recommended 21 to 28 days for ESGD treatment.^19^, ^20^ Continuation of treatment for an additional 28 days in clinical cases is dependent on follow‐up gastroscopy results, but is often necessary for cases of equine glandular gastric disease (EGGD).^20^ In human medicine, treatment with omeprazole for <6 weeks, 6 weeks—1 year or >1 year is defined as short‐, medium‐ and long‐term treatment, respectively.^9^

The occurrence of hypergastrinemia based on the 2.5‐fold increase in serum gastrin concentration in response to omeprazole treatment identified in our study is consistent with a previous study in horses in which serum gastrin concentrations doubled within 14 days in omeprazole treated horses.^2^ Hypergastrinemia in response to omeprazole treatment has been well reported in studies of humans,^9^, ^10^, ^11^, ^12^, ^13^, ^21^, ^22^, ^23^ in whom serum gastrin concentrations increase within 5 days of starting treatment.^6^ Hence, hypergastrinemia with omeprazole treatment in horses is comparable to what is observed in humans, reflecting the increase in intragastric pH with PPI treatment and the consequent loss of negative feedback on gastrin production.^1^ Serum gastrin concentrations during the period of omeprazole treatment did not continue to increase beyond the initial increase seen by day 7. Studies in humans have shown a similar pattern, where serum gastrin concentrations did not continue to increase over the treatment period.^12^, ^21^

A rapid reccurrence of ESGD in 83% of horses within 3 days of omeprazole withdrawal has been reported.^14^ In humans, the occurrence of rebound symptoms and reccurrence of gastric disease have been observed after omeprazole withdrawal, considered to be the result of hypergastrinemia and RGH.^1^ Increased serum gastrin concentration in response to omeprazole treatment observed in our study might contribute to the short‐term development of RGH and help explain the rapid reccurrence of ESGD.^14^ However, further investigation of serum gastrin concentrations each day after omeprazole withdrawal using concurrent gastroscopy is required to determine this outcome.

In contrast, serum CgA concentrations did not clearly increase over the study period. In humans, gastrin has been shown to have trophic effects on ECL cells and increased serum gastrin concentration (107 [12‐800] ng/L),^9^ higher than reported in our study, during omeprazole treatment increased ECL cell density, leading to increased acid secretory capacity after withdrawal of omeprazole treatment.^1^, ^8^, ^9^ Gastric biopsies in humans have identified a positive correlation between increased serum CgA concentrations and ECL cell hyperplasia in response to PPI treatment in patients whose treatment ranged from 6 weeks to >1 year.^9^ In healthy human patients, increased serum gastrin and CgA concentrations have been identified within 8 weeks of PPI administration, with serum CgA concentrations remaining higher than baseline 1 to 4 weeks after discontinuation of treatment.^8^, ^12^ A positive association between exogenous doses of gastrin and ECL cell hyperplasia also has been established in rats.^5^ These effects were not clearly observed in our study, suggesting that the ECL response to hypergastrinemia in horses might differ from that of other species. If true, horses might be less predisposed to RGH than other species.

Our study identified that increased serum gastrin concentrations did not persist after the discontinuation of omeprazole treatment, with a return of serum gastrin concentrations to baseline within 2 to 4 days of administration of the last dose of omeprazole. Studies in humans have identified wide variation in the return of serum gastrin concentrations to baseline, ranging from 2 days^21^, ^24^ to 4 weeks.^12^ Daily blood samples after omeprazole discontinuation are required to more accurately determine the return of serum gastrin concentrations to baseline in the horse.

Considering the rapid return of serum gastrin concentrations to baseline and lack of clear effect on serum CgA concentrations, we propose that our findings do not support the use of tapering periods after medium‐duration omeprazole treatment in the horse. Instead, management practices targeted at decreasing ESGD risk, such as rest or decreased exercise and increased roughage intake, ideally including alfalfa hay,^20^ should be optimized in the 2 to 4 days after discontinuation of omeprazole. Whether longer treatment durations would alter these results and recommendations is unknown but warrants further evaluation.

The main limitations our study were the lack of validation of the CgA ELISA kit for use in horses, 1 or 3 days without omeprazole treatment mid‐protocol, and lack of control horses. The results of the CgA ELISA assays indicated high coefficients of variation for both inter‐ and intra‐assay comparisons, indicating that the accuracy of the results might be limited. Validation of CgA testing in horses and gastric biopsies to evaluate ECL cell mass are recommended to determine the effects of omeprazole treatment on ECL cell density and RGH in horses, as has been validated in humans.^7^, ^9^ The study population was subject to recommended omeprazole withholding periods,^14^ resulting in inconsistent administration of omeprazole that might have influenced serum gastrin and CgA concentrations. However, no effect was seen on the withholding period during the treatment window (no difference between sample days 28 and 35) and we propose that the application of RWPs reflects clinical use of omeprazole in the population studied. Further study of uninterrupted administration over a similar period might be useful to provide data reflective of other populations in which continuous administration is used.

Although baseline serum measurements were taken for each horse, having control horses that were not treated with omeprazole within the same training environment might have been advantageous. Furthermore, serum gastrin concentrations are higher in horses in training than in those at rest and higher post‐feeding in horses in training, suggesting exercise and feeding to be factors affecting serum gastrin concentrations in addition to the influence of omeprazole treatment.^25^, ^26^ Our study design controlled for exercise, feeding and timing of sample collection, while determining that serum gastrin concentrations increased with omeprazole treatment and returned to baseline after omeprazole discontinuation. Gastroscopy was performed in each horse, but these findings were reported in another study.^14^

## 5 CONCLUSIONS

We confirmed rapid development (within 7 days) of hypergastrinemia in Thoroughbred racehorses treated with omeprazole and the subsequent return of serum gastrin concentrations to baseline within 2 to 4 days of discontinuation. No clear effect of omeprazole treatment on serum CgA concentrations was observed, but accuracy of the CgA results is limited. These findings do not support tapering of omeprazole treatment after medium‐term administration, but suggest that management practices targeted at decreasing ESGD risk be optimized in the 2 to 4 days after discontinuation of omeprazole treatment.

## CONFLICT OF INTEREST DECLARATION

Dr Ben Sykes has active consultancies with Kelato Animal Health Australia, Equestra/Troy Australia, and Abbey Labs Australia, all of which have products that support horses diagnosed or at risk of equine gastric ulcer disease. No other authors declare a conflict of interest.

## OFF‐LABEL ANTIMICROBIAL DECLARATION

Authors declare no off‐label use of antimicrobials.

## INSTITUTIONAL ANIMAL CARE AND USE COMMITTEE (IACUC) OR OTHER APPROVAL DECLARATION

Approved by the Ethics Committee of the Hong Kong Jockey Club, protocol code ERC/031/2021.

## HUMAN ETHICS APPROVAL DECLARATION

Authors declare human ethics approval was not needed for this study.
